# Mechanisms, predictors and clinical impact of early neurological deterioration: the protocol of the Trondheim early neurological deterioration study

**DOI:** 10.1186/s12883-014-0201-4

**Published:** 2014-10-28

**Authors:** Bernt Harald Helleberg, Hanne Ellekjær, Gitta Rohweder, Bent Indredavik

**Affiliations:** Research group for Geriatrics, Stroke and Movement (GeMS), Department of Neuroscience (INM), Norwegian University of Science and Technology, N-7489 Trondheim, Norway; Stroke Unit, Department of Internal Medicine, St. Olavs Hospital, University Hospital in Trondheim, N-7006 Trondheim, Norway

**Keywords:** Early neurological deterioration, Stroke progression, Stroke scales, Acute ischemic stroke, Clinical classification

## Abstract

**Background:**

10-40% of patients with acute ischemic stroke (AIS) suffer an early neurological deterioration (END), which may influence their long term prognosis. Multiple definitions of END exist, even in recently published papers. In the search for causes, various biochemical, clinical, and imaging markers have been found to be associated to END after AIS in some but not in other studies.

The primary aim of this study is to assess the contribution of END to functional level at 3 months post stroke measured by modified Rankin Scale (mRS). Secondary aims are to identify factors and mechanisms associated with END and to define the prevalence, degree and timing of END in relation to stroke onset, and to compare Scandinavian Stroke Scale (SSS) and National Institute of Health Stroke Scale (NIHSS) based END-definitions.

We hypothesized that END detected by changes in NIHSS and SSS (according to previously published criteria) at a threshold of 2 points indicate worsened prognosis, and that SSS is not inferior to NIHSS in predicting such a change. We further hypothesized that clinical deterioration has several causes, including impaired physiological homeostasis, vascular pathology, local effects and reactions secondary to the ischemic lesion, along with biochemical disturbances.

**Methods:**

Single-centre prospective observational study.

Participants: Previously at home-dwelling patients admitted to our stroke unit within 24 hours after ictus of AIS are included into the study, and followed for 3 months. They are managed according to current procedures and national guidelines. A total of 368 patients are included by the end of the enrolment period (December 31^st^ 2013), and the material will be opened for analysis by June 30^th^ 2014.

Frequent neurological assessments, continuous monitoring, and repeated imaging and blood samples are performed in all patients in order to test the hypotheses.

**Discussion:**

Strengths and weaknesses of our approach, along with reasons for the methods chosen in this study are discussed.

## Background

Almost 60 years ago, a clinical course of stroke with increasing severity of stroke symptoms after the onset was described [1-3]. Terminology, scales used for clinical assessment, time frames and threshold for clinical significance have varied between studies on patients suffering what is now most commonly termed early neurological deterioration (END) [4-9].

END after acute ischemic stroke (AIS) has been found to be related to case fatality and reduced functional outcome [7,8,10-12], and has been associated with various findings, subsequently generating various hypotheses.

A relation to thrombus extension has been suggested. A study conducted in 1983 compared conventional angiographic findings on admission to control angiographs in 15 ischemic stroke patients with neurological deterioration. The findings indicated increased thrombus extension in 8 patients and occlusion of a collateral vessel in 4 patients [13]. However, such findings have not been verified in more recent studies using computer tomography (CT) or magnetic resonance imaging (MRI) [14].

The literature includes studies where a positive correlation has been found between END and various factors, including age, previous diagnosis of diabetes and coronary heart disease, greater extent of hypodensity on initial CT scan [8], presence of a hyperdense media sign [8], of MCA flow velocity changes, poor cerebral hemodynamic reserve [15,16], increased stroke severity [17,18], presence of an internal carotid artery (ICA) or middle cerebral artery (MCA) occlusion [11], degree of carotid stenosis [18], and with levels of plasma glucose [19-21], ferritin [19], excitotoxic aminoacids in CSF and blood [22], pro-inflammatory cytokines [21], nitric oxide in CSF [23], and D-dimer [24] and with blood pressure values [7,18,25], and body temperature [11].

Other studies have found a negative correlation between END and blood pressure [6] and anti-inflammatory cytokines [26].

High and low triglyceride values have been found to be associated with haemorrhagic transformation, and low triglyceride values have been found to be associated with an increased risk of END [27].

Hypoxia is another suggested marker for END, which may act both directly and via a steal phenomenon [28].

Stroke subtypes may have different risks of END. Lacunar infarctions have been found to be associated with END when compared to other ischemic stroke subtypes with similar severity [29,30].

After thrombectomy and thrombolysis, other mechanisms may additionally be at play. After initial recanalization, reocclusion may occur and lead to END in some patients [31], while new infarctions are found in areas which are initially mildly hypoperfused in other patients. Thus impaired clearance of emboli in hypoperfused tissue distal to stenoses has been hypothesised [32,33]. Furthermore, parenchymal haemorrhagic transformation may cause END, and occurs more frequently in patients receiving recanalizing treatment [14]. A case of classical hyperperfusion syndrome after recanalization in a patient with END has also been reported [34].

Various models have been used to describe mechanisms leading to END, including collateral failure, clot progression, reocclusion (after initial reperfusion), recurrent stroke, cerebral oedema, hemorrhagic transformation and seizures [14], hemodynamic factors, excitotoxicity and inflammatory mechanisms [35]. There is little evidence to support any specific treatment which could reduce the impact of END in patients suffering from such deterioration or protect against END. However, stroke unit treatment is associated with a reduced risk of END and recurrent stroke [36]. Whether it also reduces the impact of END on functional level and mortality is not known. Thus, further research in the area of END is needed.

## Aims

### Primary aim

To assess the relation between END after AIS and the patient’s functional level at 3 months post stroke, including case fatality.

### Secondary aims

To define the prevalence, severity and timing of END and early deteriorating episode (EDE) in relation to AIS onset.

To identify factors and possible mechanisms associated with END and EDE after AIS.

To assess the relation between early deteriorating episode (EDE) and functional level at 3 months post stroke, including case fatality.

To compare Scandinavian stroke scale (SSS)- and NIHSS-based END-definitions.

To assess the impact on renal function and risk of contrast induced nephropathy in stroke patients examined with intravenous contrast agents.

## Methods

### Definitions

Early neurological deterioration was defined in the European progressing stroke study (EPSS) definition as any significant neurological deterioration from baseline to 72 hours [10]:

A significant neurological deterioration was primarily defined as a decrease in the SSS items score for consciousness, speech, gaze, arm, or leg by at least 2 points. Consciousness was given precedence over the other signs [10].

An alternative definition of significant neurological change was an increase in the total NIHSS score by at least 2 points.

The EPSS defined a significant deterioration between 0 and 72 hours as a neurological progression, and significant deterioration or death within 72 hours as a progressing stroke.

Early deteriorating episode (EDE) was defined as a significant neurological worsening between two consecutive assessments during the first 72 hours [10].

In this study, our definition of END is similar to that of stroke progression used by EPSS, and EDE is defined as a significant change between two consecutive assessments, in accordance with the EPSS.

New stroke was defined as a new onset of focal or neurologic deficits that could not be attributed to the presenting lesion and were consistent with World Health Organization definitions of stroke [37].

Contrast induced nephropathy (CIN) was defined as an increase from the baseline serum creatinine concentration of at least 44 μmol/L (0.5 mg/dL) or ≥25% within 48 to 72 hours after exposure to contrast media [38].

### Project context

This study is conducted within the stroke unit at St. Olav’s Hospital, the University Hospital in Trondheim, Central Norway. For stroke treatment it has a recruitment area of 230 000 inhabitants as a local hospital, serves another 100 000 for thrombolytic evaluation and -treatment, and serves a total of 700 000 inhabitants in Mid-Norway for invasive treatment, including thrombectomy. The comprehensive stroke unit has a long tradition for combined acute treatment and early mobilization [39], and offers an early supported discharge service [40]. It is organized as a unit within the Department of Internal Medicine. All patients above 60 years of age with acute stroke symptoms are admitted to this stroke unit, as long as there is treatment capacity within the unit, while younger patients routinely are admitted to the Department of Neurology.

On average, 325 patients per year have been discharged from the stroke unit with a diagnosis of ischemic stroke during the period. This number also includes patients transferred from other departments after procedural strokes, or after initial treatment in other wards in the hospital.

All patients within the study receive medical treatment and follow up according to current national guidelines and local protocol. Both emphasise the importance of evaluating the patient’s physiological homeostasis and recommend measures to restore it. In case of increasing symptoms, the local protocol recommends an infusion of ½-1L Ringer-acetate within 1–3 hours, while platelet inhibition with both Aspirin and Clopidogrel may be considered after exclusion of a haemorrhage.

Inclusion into this study should not affect length of stay or treatment decisions.

### Study design

The present study is a prospective observational study, performed in a single stroke unit from May 17^th^ 2010 to December 31^st^ 2013, comparing patients with early neurological deterioration to those with no such deterioration.

### Study population

This study intended to include consecutive patients admitted to the stroke unit with acute stroke symptoms after exclusion of haemorrhage on initial native CT examination.

The pilot study included independent patients <85 years of age, admitted within 8 hours after debut of stroke symptoms, without haemorrhage, stroke sequelae, previously known terminal illness or other condition which might confound follow-up, other final diagnosis than stroke, or contraindications to intravenous contrast agents. In the main study, patients admitted within 24 hours were included. After October 16^th^ 2010, patients were no longer excluded due to dependency, age, or contraindications to i.v. contrast agents.

Thus, after the last revision the study intended to include all patients who were

- Admitted to the stroke unit with acute stroke symptoms
- Admitted to the stroke unit within 24 hours after ictus, and are
- Previously living in their own home

The exclusion criteria were

- Previously known preexisting condition which could confound follow-up
- Consent could not be achieved
- Haemorrhage on native CT examination
- If a diagnosis other than acute ischemic stroke provided at least as good an explanation to the patient’s symptoms.
- No capacity to follow the patient according to the study protocol.

### Primary outcome

The effect of END according to the EPSS definition on level of function and case fatality at 12 weeks, measured by the modified Rankin Scale (mRS) compared to patients without END.

### Secondary outcomes

The effect on level of function and case fatality at 12 weeks, measured by mRS, of EDE compared to the patients with a stabile or improving course.

Prevalence, timing and severity of END and EDE according to both SSS and NIHSS based definitions.

Clinical, hemodynamic, haemorheological, biochemical, metabolic and vascular factors associated with END.

Initial CT and subsequent CT/MR images will be examined in order to identify a relation between END and findings on imaging, and both ASPECTS and Pc-ASPECTS scores will be used to quantify relevant findings.

The effect of END on level of function and case fatality measured by mRS for patients fulfilling the criteria of NIHSS based definitions of END.

An estimate for the minimal detectable change will be calculated for both the NIHSS and SSS, based upon previously published data and the standard deviation found in our study.

The effect in stroke patients of exposure to intravenous contrast agents due to radiological procedures on renal function.

### Data collection

#### Background data

Age, gender, level of function, medical history, and current medical treatment at admission are collected on admission and supplemented from relevant sources while the patients are hospitalized. Medical treatment at 24 hours and 7 days (or discharge, whichever is sooner) is also recorded.

#### Neurological assessments

Severity of stroke is assessed according to NIHSS, SSS and a locally adapted abbreviated SSS form, including the items for consciousness, speech, eye, arm, hand and leg motor score (Similar to the standardised nursing observations for stroke (SNOBS), only extended with the hand item), with simultaneous blood pressure and oxygen saturation measurements. Examinations according to this latter form are performed at admission and every 6^th^ hour until 48 hours, and at 60 and 72 hours. Additionally, the full SSS and NIHSS are scored at admission, after 24, 48 and 72 hours, at 7 days (or discharge, if after 72 hours and before 7 days), and 1 and 3 months. All relevant personnel responsible to perform these scores were trained in scoring according to these scales prior to the study.

A subtype classification of stroke is performed according to the Oxfordshire stroke project classification. The information is based on the information available from the score sheets, supplementary neurological examination and relevant clinical information.

#### Functional level assessments

A pre-stroke mRS is estimated from available information, and mRS is also scored at 24, 48 and 72 hours, at 7 days and 1 and 3 months. Barthel ADL index is scored at 24 hours, 7 days, 1 and 3 months.

#### Monitoring of homeostasis

A clinical status is performed at admission. Oxygen saturation, ECG, and heart rate is monitored continuously for the first 24 hours. The blood pressure is also measured hourly for the same period. Except ECG, these data are also registered at the SNOBS scorings during the first 24 hours, and blood pressure is registered at every SNOBS scoring the first 72 hours.

Temperature and glucose are measured every 6^th^ hour the first 48 hours, and at 60 and 72 hours.

#### Blood samples

During the first 72 hours, routine blood tests are taken repeatedly (Table 1).

**Table 1:** **Blood samples taken during the first 72 hours**

	**Admission**	**Inclusion (<4 h)**	**24 hours**	**48 hours**	**72 hours**
Sodium	X		X	X	X
Potassium	X		X	X	X
Creatinine	X		X	X	X
Urate			X		
Osmolality		X	X	X	X
Haemoglobin	X		X	X	X
Leucocyte count	X		X	X	X
Platelet count	X				
EVF	X				
PT-INR	X				
Fibrinogen		X		X	X
D-dimer		X		X	X
Troponin T	X		X		X
Total cholesterol			X		
HDL cholesterol			X		
LDL cholesterol			X		
Triglycerides			X		
Glucose	X		X		
HbA1c			X		
CRP	X	X	X	X	X
HS-CRP		X	X		X
Lp(a)		X			
Albumin		X			

#### Imaging

A native CT examination of the brain is performed at admission in order to exclude haemorrhage. A control examination is performed (either CT or MRI) at 24–72 hours in order to visualize the extent of ischemic damage.

When clinically relevant (e.g. patients eligible for iv. Thrombolysis or i.a. thrombectomy or suspected carotid stenosis) and not contraindicated, a brain and neck contrast-enhanced CT-angiography is performed on one of these occasions. Otherwise, carotid ultrasonography is performed.

#### Complications

Medical complications are noted if reported in the medical records.

#### Flowchart

Table 2 shows a flow chart of the examinations obtained and when.

**Table 2:** **Examinations and follow up in the Trondheim END Study**

	**Inclusion**	**24 h**	**48 h**	**72 h**	**1 w**	**4 w**	**12 w**
**Consent**	**+**						
**Blood samples**	**+**	**+**	**+**	**+**			
**Monitoring (24h;** BP, ECG, O2,respiration)	**+**	**+**					
**Glucose**	**X4**	**X4**	***X*** **2**	**X1**			
**Temperature**	**X4**	**X4**	***X*** **2**	**X1**			
**ECG**	**+**	**+**		**+**	**+**		
**SSS**	**+**	**+**	**+**	**+**	**+**	**+**	**+**
**NIHSS**	**+**	**+**	**+**	**+**	**+**	**+**	**+**
**CT**	**+**	**+**	**or +**				
**CT-angiography**	**(+)**	**(+)**	**(+)**				
**MRI**		**(+)**	**(+)**	**(+)**			
**Carotid ultrasound**		**(+)**	**(+)**				
**Modified Rankin Scale**	**Prestroke**	**+**		**+**	**+**	**+**	**+**
**SNOBS**	**+**	**+**	**+**	**+**			

+denotes when examinations etc. should be performed Xn denotes that it should be performed n times, (+) denotes that an examination should be performed, unless another optional examination is performed with success.

#### Supplementation of data

Missing data will be retrieved or estimated from medical records or, for the neurological scorings, from other scoring sheets. Scorings may also be adjusted when there is an obvious inconsistency between the scoring and other clinical information. In cases where data are missing and cannot otherwise be reliably estimated in a clinical (functional or neurological) score sheet, no change is assumed to have occurred, and the former value is continued. Thus, patients who are discharged before 72 hours will be scored in accordance to their last assessment, unless the medical records report worsening within 72 hours.

### Power and sample size estimations

Our stroke registry data indicate that approximately 500 patients are treated for ischemic stroke and intracerebral haemorrhage combined at our hospital per year. Approximately 10% of all acute stroke admissions are caused by haemorrhagic stroke. 70% of acute stroke patients are treated primarily in the stroke unit. An observational study on complications performed in our stroke unit, included 443 patients with ischemic stroke and 46 patients with haemorrhagic stroke in 16 months, yielding an average of 332 patients per 12 months. Thus, at least 300 patients were expected to be eligible for inclusion to the study per year.

From previous studies, one in six patients was expected to suffer a clinical deterioration according to current definitions. 80% power to detect a 15% increase in dependence at a significance level of 0.05 needed 600 patients. Due to the considerations above, the study was planned for inclusion of 600 patients over the course of 2 years.

### Statistics

Relevant statistics will be used for descriptive data for the population, and single- and multivariable analyses will be applied in order to find factors related to END within our material.

An ordinal logistic regression analysis will be performed in order to assess the effect of END on function on the full range of the mRS.

Minimal detectable change will be estimated for both END definitions, based on previously published data and the standard deviation within this material.

### Time plan of the study

A pilot study was performed in 2009. Inclusion in the main study was initiated on May 17^th^ 2010, and the last patient was included in December 2013. A total of 401 patients have been included into the main study, of which 39 patients are excluded. The material from another 6 patients from the pilot study was sufficiently complete to be included into this study, leaving a material of 368 patients eligible for analysis in this study. Follow up for the last patient was complete April 7^th^ 2014, and the complete data set is not open for analyses until June 30^th^ 2014.

### Consent and inclusion into the study

All patients are asked to participate on arrival and receive written information about the study and possible consequences of participation. A written consent, or witnessed informed oral consent, is obtained as soon as possible after arrival. In cases when the patients are suspected not to be able to give an informed consent, a proxy, typically the next of kin is used, and delayed informed consent or non-consent will be achieved from the patient in cases when the patient is able to make such a decision at a later time-point.

### Ethics

The study and its pilot were approved by the Regional Committee of Ethics in Medical Research (Central Norway) (REK4.2010.492 and REK4.2008.2278).

Although monitoring itself is not harmful, the patients may not be mobilized as early and to the extent they would outside of this study. A limitation of early mobilization may be harmful, although this has not been proven [41]. An informed consent was obtained from all participants according to the procedures reported above.

## Discussion

This study aims to assess both the relation between END according to different stroke scales and case fatality and level of function after 3 months, and give a broad account of association between END and potential associated markers in a large material of unselected patients.

The primary aim in this study was to assess the relation between END after AIS and the patient’s functional level at 3 months post stroke, including case fatality. This relation has been found in previous studies, with various definitions of END [7,8,10-12]. A study of the relation between END and functional level and case fatality may still be relevant. Our comprehensive stroke unit is research driven, with thorough documentation on its benefits, sustained up to 10 years after stroke, in regard to functional outcome and survival compared to control [39,42,43], and also improved functional outcome when combined with early supported discharge [40,44]. Some of the benefit of the stroke unit treatment may be due to a reduction of complications, including stroke progression and recurrent stroke, which may be due to comprehensive implementation of various measures to secure physiological homeostasis [36]. Data from our stroke unit on complications during 2002–2003 in unselected stroke patients found END in 18.9% of the patients, which is rather low in a patient material with intracerebral haemorrhage and AIS combined [37]. Thus, there are reasons to believe that the incidence of END as well as the effect of END on functional outcome and mortality may have been reduced due to improvements in treatment.

This study is based upon our day-to-day-treatment and follow-up in the stroke unit. Thus, any relevant findings from this study may be clinically applicable. Further, unlike most studies in this area, this study applies repeated neurological scorings, ideally every 6^th^ hour the first 48 hours, and every 12^th^ until 72 hours, which may allow measures to be taken sufficiently early for these to be efficient. This study may also quantify at which level deterioration can reliably be detected, without causing the excessive work-up expected by others [4].

Some of the choices made in the design and conduction of the study constitute limitations, and the choices may need to be justified.

Mechanisms that have been used to explain END include collateral failure, clot progression, reocclusion (after initial reperfusion), recurrent stroke, cerebral oedema, haemorrhagic transformation and seizures [14], hemodynamic factors, excitotoxicity and inflammatory mechanisms [35]. These mechanisms may not exclude each other, but could be seen as supplementary, and may be in play in various degrees in different patients. The penumbra may increase in response to systemic hypotension, intracranial hypertension, vasogenic oedema or hyperglycemia [45], hypoxia or hypercapnia [28] and (re-)occlusion. The positive correlation between risk of END and degree of carotid stenosis [18] may be seen as a marker for susceptibility for hypotension, and thus END, as also reduced cerebral hemodynamic reserve [15]. Hyper- and hypoglycaemia [46] and fever [47] affect the metabolism in the tissue in a way that may threaten the hypoperfused tissue. However, not all mechanisms proven to be relevant after a stroke are limited to the penumbra [33]. We have established an examination programme in order to find factors and mechanisms related to END, with special emphasis on physiological homeostasis and hemodynamic factors. Further, seizures are noted when observed in clinical practice, new stroke may be detected by clinical observation or imaging, and cerebral oedema and haemorrhagic transformation should be detected by imaging. Some blood tests and CSF tests which have been associated with END have been left out of the program due to cost and infeasibility. Thus the exitotoxicity factors and some of the immunological factors cannot be explored within our study. However, in addition to markers present on admission, we believe that our protocol allows us to identify transient risk factors for deterioration to a greater extent than previous studies, as our scorings give an opportunity to identify when changes in neurological function occur, and the large battery of tests within our protocol may help identify causes for the deteriorations that occur.

The choice of definition of END, in studies as well as in repeated clinical evaluation, deserves a thorough discussion. In dealing with END definitions, as in other approaches to define meaningful clinical change, both an anchor-based approach and a distribution based approach should be applied [48]. Obviously, the first concern is that the change found is clinically important (minimal important change). One acceptable approach is anchoring the definitions in endpoints, which has been done with various SSS-based, NIHSS-based and CNS-based definitions, which have been found to be related to a poor outcome [7,8,10-12].

In the EPSS validation, the relation between functional level and inclusion or exclusion of different items, and also the CNS definition, was found to be in favour of the EPSS definition, and was again validated when tested against two other definitions [10,12]. To our knowledge, no such comparison exists between the NIHSS and the SSS.

As the EPSS definition was established when the study was planned, and there was no agreement on NIHSS definition [4,11,35], this study was planned with the EPSS definition as main definition of END, which is well anchored in clinical endpoints.

Although the distribution based approach to clinical important change has been much neglected within this area, it has its strengths in taking the error of the measurements in account, which is well expressed in the concept of minimal detectable change (MDC) [48]. This may be calculated from previously published data, supplemented with our data in the case of SSS, but in the case of the EPSS definition, an MDC for the full scale or relevant items may have limited value.

Initially, the study protocol included a contrast enhanced CT-angiographic examinations of all patients on admission and repeated at a routine control examination after 36–72 hours, in addition to be repeated in case of progression, in order to detect a possible association between thrombus progression and END. Introduction of perfusion examination at some or all these examinations was suggested. All such contrast-enhanced examinations may lead to increased risk of contrast induced nephropathy (CIN), in addition to risk associated with increased radiation. The most commonly used definition of CIN is an increase from the baseline serum creatinine concentration of at least 0.5 mg/dL or at least 25% within 48 to 72 hours after exposure to contrast media, and has been reported in 2% of patients in general population, increasing to as much as 50% of patients with multiple risk factors, and depends on the volume of contrast given [38]. Patients with previously known creatinine > 130 μmol/L (>1,5 mg/dL), age >85 years of age, or known adverse reactions to contrast agents would not be examined routinely with CT-angiography (and subsequently CT-perfusion if admitted within 8 hours), as the risk was considered greater than a possible benefit. Thus, they initially were not included into the study. After an early revision, these patients were examined with carotid ultrasound. After a change in the clinical protocol, only patients eligible for thrombectomy would be examined with a CT-angiography for an acute assessment, and other patients only on clinical indication. CT-perfusion was abandoned due to time loss. This fact does not change the fact that patients have been exposed to risk of CIN. This may be justified by findings relevant for clinical treatment. As creatinine has been repeatedly taken, this allows an estimation of this risk in patients selected according to these criteria, and thus, an estimation of the risk of CIN in patients exposed, can be quantified.

Despite changes in inclusion criteria, far less than the expected 300 patients per year were included. Due to limited capacity in the stroke unit, some patients were not primarily admitted, and were thus not eligible for the study. Of the patients admitted to the stroke unit, an important proportion was not included into the study. Possible causes may be uncertainty about the diagnosis, and that medical treatment and care had to be given priority over inclusion in this study. Selection bias can be suspected. Patients receiving thrombolytic therapy are always admitted to the stroke unit, and are followed according to the study protocol, and may thus be more prone to be included into the study. Patients with more subtle stroke symptoms may less likely be included, as mild stroke symptoms may be overlooked and symptoms from posterior circulation may be less specific for stroke, and thus believed to not be caused by other. These changes may give inadequate estimates of incidence of the stroke types and severity of stroke, and thus may give inadequate estimates for incidence of progression and prognosis after stroke. As some information concerning stroke severity exists in our stroke registry, the effect of a possible selection bias may be discussed.

Despite these limitations, we believe that this study will contribute to knowledge within this area.

## Abbreviations

AIS: Acute ischemic stroke
BP: Blood pressure
CDM: Clinical-diffusion mismatch
CIN: Contrast induced nephropathy
CNS: Canadian neurological stroke scale
CSF: Cerebrospinal fluid
CT: X-ray computed tomography
EDE: Early deteriorating episode
END: Early neurological deterioration
EPSS: European progressing stroke study
EVF: Erythrocyte volume fraction
HDL: High density lipoprotein
HbA1c: Glycated haemoglobin
HS-CRP: High sensitivity C-reactive protein
ICA: Internal carotid artery
LDL: Low density lipoprotein
LP(a): Lipoprotein(a)
MCA: Middle cerebral artery
MRI: Magnetic resonance imaging
mRS: modified Rankin scale.
NIHSS: National Institute of Health stroke scale
PT-INR: Prothrombin time derived international normalised ratio.
SNOBS: Standardised nursing observations for stroke
SSS: Scandinavian stroke scale
